# Supplementary material for: Interferon beta for the treatment of multiple sclerosis in the Campania Region of Italy: Merging the real-life to routinely collected healthcare data
Source: PLoS One. 2021 Sep 29;16(9):e0258017. doi: 10.1371/journal.pone.0258017 (PMC8480611; doi:10.1371/journal.pone.0258017)
Supplement: Attachment — Submitted filename: Response to Reviewers.docx [file pone.0258017.s001.docx]

**Reviewer #1**

**In this interesting work, the authors found that differences between interferon beta formulations in Campania region were related to adherence and healthcare resource utilization, by merging routinely-collected healthcare data to a clinical registry. They showed that this innovative approach for data analysis is feasible and can therefore be largely used in future research. The work is clear and well written, I only have some minor comments.**

We thank the reviewer for his/her positive feedback.

**In this cohort of patients, the risk of discontinuation was the highest in patients treated with Betaferon®/Extavia®, which were those with higher EDSS at baseline. Can the authors provide any reassurance that the worse disability at the beginning of the disease did not impact the analysis?**

We have adjusted all statistical models for baseline EDSS, as reported in the statistical methods. We have now also highlighted this in the limitations’ section of the Discussion:

*“Our study suffers from different limitations, mostly arising from …differences in baseline* *characteristics, that we tried to mitigate by using covariates in the statistical model”.*

**Have the authors explored whether different interferon beta formulations influence the risk of time to switch to a second line MS treatment and time to conversion to secondary progressive disease course, if data after 2019 were available?**

Unfortunately data after 2019 were not available and, as also suggested by the Reviewer, we decided not to evaluate conversion to secondary progressive disease course.

On the contrary, we have now evaluated the time to second-line DMT as shown in the table below.

| **Switch to second line DMT** | **Coeff.** | **P.value** | **95% Conf.** | **Interval** |
| --- | --- | --- | --- | --- |
| **Rebif®** | *Reference* | | | |
| **Avonex®** | -0.589 | 0.006 | -1.007 | -0.172 |
| **Plegridy®** | 0.233 | 0.475 | -0.406 | 0.872 |
| **Betaferon®/Extavia®** | 0.057 | 0.770 | -0.326 | 0.441 |

Overall, results mirror what we have already found in relation to ARR, and, thus, do not seem to add much to the paper. As such, we have decided not to include this analysis in the main body of the manuscript which actually focuses on clinical outcomes (relapses and disability).

**Disease duration of MS patients at baseline should be added to Table 1.**

We have now added disease duration to **Table 1**.

**Reviewer #2**

**In this article Moccia and colleagues describe differences in clinical outcomes, healthcare resource utilization and costs between interferon beta formulations for MS, merging a clinical registry to routinely-collected healthcare data. Methods are sound, the statistical analysis has been performed appropriately and rigorously.**

We thank the reviewer for his/her positive feedback.

**There is only a minor concern. Regarding the sentence: “Looking at clinical outcomes, rates of relapses and EDSS progression were lower than studies run on older cohorts.” not is clear if the authors mean older age cohorts or previous cohort. It should be clarified.**

As suggested, we have revised to “previous cohort”.

**Furthermore, in the opinion of the authors, the introduction of the new diagnostic criteria in the recent years, could have influenced the results of the study and the differences with older age cohorts? (PMID: 30419509).**

We thank the Reviewer for suggesting this, and have now revised the following sentences in the **Discussion** (along with reference):

“*Looking at clinical outcomes, rates of relapses and disability progression (estimated using a roving EDSS as reference) were lower than studies run on previous cohorts, possibly also as a consequence of new diagnostic criteria*”
